# The Gut-Liver-Brain Axis: From the Head to the Feet

**DOI:** 10.3390/ijms242115662

**Published:** 2023-10-27

**Authors:** Mauro Giuffrè, Rita Moretti

**Affiliations:** 1 Department of Internal Medicine (Digestive Diseases), Yale School of Medicine, Yale University, New Haven, CT 06510, USA; 2 Department of Medical, Surgical and Health Sciences, University of Trieste, 34149 Trieste, Italy; moretti@units.it

The gut-liver-brain axis, a multifaceted network of communication, intricately connects the enteric, hepatic, and central nervous systems [1,2]. One crucial aspect of this complex interaction is the impact of the brain on the functions of the intestines and liver, specifically in relation to the modulation of immune cell activities [3]. On the other hand, the gastrointestinal tract and liver have substantial impacts on cognitive function and overall mental health, primarily by influencing the composition of microbiota and regulating innate immune responses [2].

Most of the initial studies on the gut-liver-brain axis were focused on understating its potential role in functional gastrointestinal disorders (such as irritable bowel syndrome), given the neuroemotional stress that function as a trigger for such disorders [4,5,6,7,8,9,10,11,12,13,14,15]. However, more recent studies have investigated the potential role of the gut-liver-brain axis in other gastrointestinal disorders such as celiac disease or inflammatory bowel disease [16,17]. An imbalance or disruption in the gut-liver-brain axis has also been linked to several neurological conditions, including hepatic encephalopathy, delirium, autism, attention deficit hyperactivity disorder, depression, Alzheimer’s and Parkinsons’s disease [18,19,20,21,22,23,24,25]. However, the exact mechanisms behind this connection remain mostly elusive, but researchers propose links to the within-host evolution of gut microbiota, metabolic dysfunctions, and systemic inflammation caused by gut dysbiosis.

This Special Issue has cast its net wide, encompassing a spectrum of topics from foundational biological sciences to the avant-garde realms of machine learning and artificial intelligence applied to gut microbiota research [23]. In particular, venturing into the domain of neurodegenerative diseases, it was explored how colon inflammation may be linked to neuroinflammation and neurodegeneration, or how the gut microbiota has a role in safeguarding cognitive prowess, facilitated through the parasympathetic nervous system.

In light of these significant findings and considerations, we express our profound gratitude and are deeply honored to have spearheaded this Special Issue. Our heartfelt appreciation extends to all contributing authors for entrusting their groundbreaking research to the International Journal of Medical Sciences.
